# Just Transition Toward Clean Energy Access With Focus on Lead‐Based Perovskite Solar Cell Technology: Lessons, Experiences, and Future Perspectives

**DOI:** 10.1002/gch2.70135

**Published:** 2026-07-31

**Authors:** Benjamin K. Korir, George G. Njema, Mojeed A. Agoro, Thembinkosi Donald Malevu, Joshua K. Kibet

**Affiliations:** ^1^ Department of Chemistry Egerton University Njoro Kenya; ^2^ Institute of Technology University of Fort Hare Alice South Africa; ^3^ Material Science, Innovation and Modelling (MaSIM) Research Focus Area Department of Physics North‐West University Mahikeng South Africa

**Keywords:** circular economy, energy poverty, environmental impacts, perovskite solar cells, social acceptance

## Abstract

Energy poverty remains a critical barrier to sustainable development, particularly in emerging and developing economies. Whereas solar technologies enjoy a positive public perception compared to fossil fuel infrastructure, the phenomenon of ‘green first’ or support for renewable energy can mislead government officials or policymakers into believing that social acceptance is not a key issue when deploying innovative renewable energy projects. In spite of technological advances that have progressed in the field of perovskite solar cells (PSCs), critical ethical and environmental problems surrounding their application remain unresolved. Although, significant progress with PSCs, attention has focused on performance and scalability; few studies have integrated PSC toxicity, recycling, and environmental impacts within the energy justice framework applied to sub‐Saharan Africa, Indo‐Pacific regions, Latin America and other parts of the world. This review synthesizes evidence‐based PSC environmental risks, recycling feasibility, and circular economy models, and links to potential impacts on equitable energy access in the context of environmental vulnerability and eco‐safe resource constraints. The key findings show that while PSCs can dramatically reduce the cost of decentralised solar energy and enhance energy access, they can also support successful transitions that achieve the procedural, recognition, and equitable distribution and inclusivity necessary for energy justice globally.

## Graphical abstract

To intentionally mitigate energy poverty in underserved communities, advanced perovskite energy technologies such as lead‐based perovskite solar cells are necessary. However, in future deployment of these materials, energy justice, equity, inclusivity and environmental sustainability must be taken into account. Just energy transition has therefore, become an increasingly urgent priority for policymakers, social scientists, and community activists.

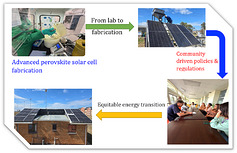

## 1 Introduction

Global energy demand continues to rise, driving rapid advances in renewable energy technologies. Regions such as sub‐Saharan Africa, Indo‐Pacific, Latin America and other parts of the world are poised to contribute significantly to primary consumption due to their rising living standards and decreasing energy poverty [1]. With climate change making energy systems based on fossil fuels progressively unsustainable and the global need to reduce its reliance on fossil fuels, the world has developed a two‐fold moral and environmental imperative for ensuring that the 675 million without access to electricity are not left behind and actively encouraging the replacement of the current carbon‐based systems [2]. This principle, embodied in the idea of a just transition (JT), envisages that the green energy revolution must be inclusive, equitable, and restorative, and must generate opportunities rather than reproduce past patterns of exclusion [1]. Photovoltaic solar technologies offer notable potential for JT, particularly in Africa where there is abundant and easily accessible solar energy resources [1]. Similarly, energy sovereignty in the context of perovskite solar cells (PSCs) refers to the capacity of individuals, communities, or countries to produce, control, and use renewable energy without depending on centralised grids or imported power [3]. PSC technologies can support the operation of decentralised energy solutions by adding local energy storage or by wirelessly transferring power via a small coil placed above flexible or lightweight PSCs. Decentralised energy solutions allow households and communities to generate electricity at the point of need, enhance energy security, reduce vulnerability to grid outages, and support sustainable development.

**FIGURE 1:**
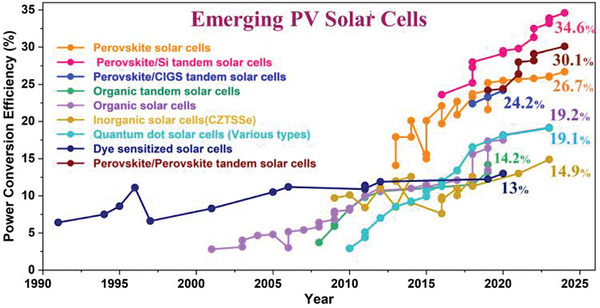
Latest advancements in emerging photovoltaic solar cells, with data sourced from NREL. Reproduced with permission [7]. Copyright 2024, Royal Society of Chemistry.

The attractive features of PSCs are driven by their low‐cost fabrication and compatibility with indoor and off‐grid applications [4]. Furthermore, ongoing advances in device efficiency and stability, along with the development of lead‐free compositions, are improving the accessibility of clean, affordable, and scalable solar energy, particularly in regions with limited energy infrastructure. According to the National Renewable Energy Laboratory (NREL), innovative solar energy projects deliver high power output at low cost, with rapid efficiency improvements recorded for perovskite‐based solar cells (cf. Figure 1). Perovskite/silicon tandem devices are expected to surpass 34% efficiency, reflecting their strong potential for next‐generation photovoltaic applications [5]. Single‐junction perovskites and all‐perovskite/perovskite tandem architectures have also demonstrated significant improvements observed in dye‐sensitised and organic solar cells. Generally, new photovoltaic technologies, such as quantum dots and inorganic Copper Zinc Tin Sulfide Selenide (CZTSSe) solar cells, are not only steadily increasing their efficiency but also have high potential for use in next‐generation solar energy applications. It is worth noting that NREL's efficiency limits capture only the technical potential; energy justice requires a rigorous examination of environmental burdens (toxicity distribution), governance (participation in decision‐making), social outcomes (poverty reduction), and economic structures (benefit sharing and cost reduction) [6]. Besides, energy justice cannot be pursued without technically viable technologies. NREL efficiency benchmarks have demonstrated the remarkable potential of single‐junction (>25%) and tandem (>33%) perovskite technologies, showing promise for addressing energy poverty (cf. Figure 1) [7].

The success of renewable energy adoption and deployment depends on the regulatory landscape and serves as a fundamental driver in achieving the ambitious United Nations Sustainable Development Goals (SDGs). PSC technology directly supports SDG #7 on affordable and clean energy and SDG #10 on social equity [8]. It also supports SDG #11 by providing sustainable urban infrastructure compatible with lightweight, flexible, and building‐integrated designs. However, unmanaged lead risks pose a threat to SDG #3 on health and well‐being and SDG #6 on clean water, particularly in rural and peri‐urban environments [8]. At present, significant gaps remain between laboratory achievements and the practical implementation of perovskite solar technologies. The encapsulation strategies have been tested under controlled conditions, but very little is known about the perovskite module in actual environments such as hail, fire, flooding, or improper disposal, especially across different microclimates. The effects of humidity in the tropics, oceanic coastal air, and acidic or alkaline soil can be drastic for the release of lead and its transport into the environment. Similarly, recycling has focused on material recovery rather than on developing inclusivity and locally viable processes for collecting and processing materials in low‐infrastructure regions. Despite growing optimism about their potential to bridge the energy access, little is known about their impact and performance in real‐world low‐income settings [9]. Socioeconomic adoption is often impeded by institutional capacity, affordability barriers, and mismatched policy frameworks [10]. These gaps are also consequential in emerging applications such as climate‐smart agricultural activities in line with SDG #2 on zero hunger. Contamination of crops, water, and soil systems is not only a technical issue but also a form of injustice, as most powerless communities are resource‐poor and most vulnerable to environmental pollution and its effects.

In this roadmap, PSC technology can scale more rapidly to meet climate mitigation targets when critical interventions, such as developing key governance structure across the solar energy deployment value chain and life‐cycle challenges are resolved. This study is motivated by the following three energy justice dimensions: (1) environmental justice, which is subject to the environmental and health hazards posed by lead exposure in lead‐based PSCs; (2) procedural justice, reflected in decisions about the development and deployment of PSCs and; (3) restorative justice, which involves repairing the harm if it occurs. In this regard, lead is not only a materials‐engineering problem to be addressed through improved encapsulation but also a governance problem that requires remediation through policy, regulatory oversight, community engagement, and circular‐economy design strategies. According to Arteaga et al. [11], PSC technology is projected to experience significant growth, with notable advances in material stability and efficiency, which is anticipated to broaden its adoption. Nevertheless, the equitable shift toward clean energy should be supported not only by efficient solar technologies but also by careful life‐cycle assessment (LCA) and strong policy support [12]. To enhance electricity availability, especially in off‐grid or underserved areas, PSC technology based on lead plays a key role in enhancing energy security and meeting renewable energy targets [13]. To this end, the analysis of PSC deployment using the JT matrix can inform future information‐based governance and regulatory initiatives, revealing synergies that undermine dichotomies between profits versus people and jobs versus environment in climate mitigation strategies [14].

## 2 Methodology

This review applied the Preferred Reporting Items for Systematic Reviews and Meta‐Analysis (PRISMA) guidelines to ensure a rigorous, systematic, and transparent process [15]. Herein, systematically collected reviews and studies concerning just transitions in renewable energy, published from 2012 to 2026 in scientific and policy databases such as IEEE Xplore, MDPI, ScienceDirect, Scopus, Google Scholar, and repositories of the World Health Organisation and the United Nations. The keywords used in the literature search were: “perovskite solar cells,” “lead toxicity,” “stability,” “encapsulation,” “life cycle assessment,” “energy justice” and “clean energy transition”. This first step was intentionally inclusive, capturing studies from materials science, environmental engineering, toxicology, and policy. The selection of scholarly materials from the 2010s marked an era during which PSC technology experienced rapid efficiency gains, rising from 3% to record‐breaking certified laboratory efficiencies > 25% [16]. Also, the 2010s saw the rise of advanced metering infrastructure (AMI), which became a springboard for scaling smart cities and microgrids [17]. Subsequently, the 2020s were marked by the rise of AI algorithms relevant to the clean energy transition and a shift toward commercialisation and durability projections [18]. Over the past 15 years, the concept of energy transition accelerated, and researchers, social scientists, and policymakers realised that decarbonisation cannot ignore the socio‐economic impacts of these innovative projects [19]. Following this context, a literature search was performed using Boolean “OR” and “AND” operators between main terms and keywords. The comprehensive literature reviews conducted using Boolean operators are a well‐established practice that has been employed in previous studies [15].

**FIGURE 2:**
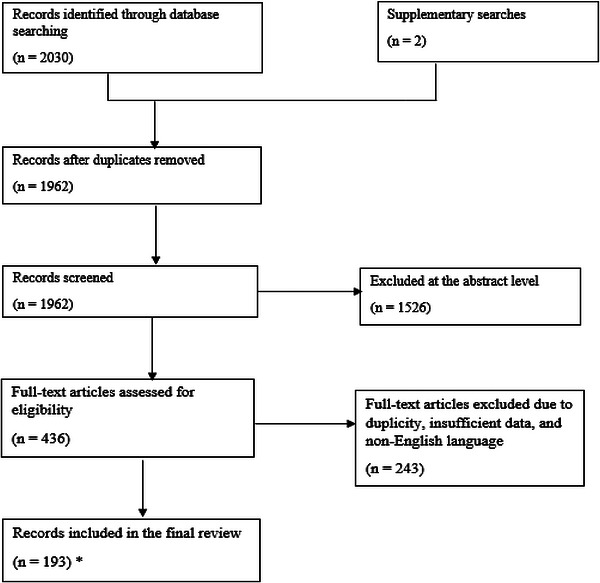
Flow chart methodology used in this review.

The selected studies focused specifically on lead degradation, environmental risk, encapsulation strategies, and life‐cycle assessment (LCA) metrics relevant to commercialization. Each selected article was systematically categorized into key themes, including device efficiency and stability, environmental risk, mitigation strategies, and end‐of‐life management. Further to technical evaluation, the review incorporated an energy justice framework to assess whether lead‐based PSCs can support a fair and sustainable clean energy transition. The analysis considered distributive justice (who benefits and who bears environmental risks), procedural justice (community involvement in decision‐making), and restorative justice (mechanisms for addressing contamination and environmental harm). Ultimately, the framework was applied to practical deployment scenarios, including urban building‐integrated photovoltaics, off‐grid rural electrification, and agrivoltaics greenhouse systems. Reviews and original papers were manually excluded if they were irrelevant to perovskite technology, and instead focused on general transitions in renewable energy. Non‐English articles were also manually excluded. The search results were imported into EndNote X8 (Clarivate Analytics, USA). Figure 2 presents a flowchart summary of the methodology used.

## 3 Overview of Industrial Commercialization of Perovskite Solar Cells for Just Energy Transitions

The rapid development of PSCs has positioned them among the most promising photovoltaic technologies, driven by remarkable efficiencies, scalable fabrication methods, and strong economic viability [12]. Perovskite‐based materials have shown rapid performance improvements, culminating in a record power conversion efficiency of 26.7%, up from 3.81%, in just a few years [16]. However, most effective PSCs incorporate lead‐based perovskites. The current efficiency of such cells is worth mentioning, and its current value surpasses that of other existing thin‐film photovoltaic technologies such as Copper Indium Gallium Selenide (CIGS) (23.6%), Cadmium Telluride (CdTe) (23.1%), and thin‐film silicon (21.2%), according to the National Renewable Energy Laboratory (https://www.nrel.gov/) accessed February 13, 2026 [16]. More significantly, PSCs offer attractive approaches to manufacturing: they can be made at low temperatures via solution processing, printed onto thin, flexible substrates, and designed to perform exceptionally well in low‐light conditions [20]. These characteristics make PSCs suitable for installing photovoltaics on buildings, for portable energy devices, and for electrifying rural areas, i.e., regions where energy poverty is greatest. Hypothetically, the democratisation of electricity with PSCs would transform off‐grid communities by enabling cheap, locally deployed solar energy [21]. Although lead‐free alternatives exist, none are as stable or as of high‐performance as their lead‐based counterparts [16]. The possibility of using lead‐containing solar modules in certain regions poses a grave threat to the environment and harms human health due to inadequate end‐of‐life (EoL) management protocol [22]. The toxicity of lead raises a critical concern as lead‐based perovskites stride toward commercialisation: can technology based on a hazardous material serve justice, sustainability, and human wellbeing? Realising full potential for equitable energy access requires deliberate policy structures addressing ownership models, community decision‐making, and environmental governance, aspects that are not determined by technical performance [23].

**FIGURE 3:**
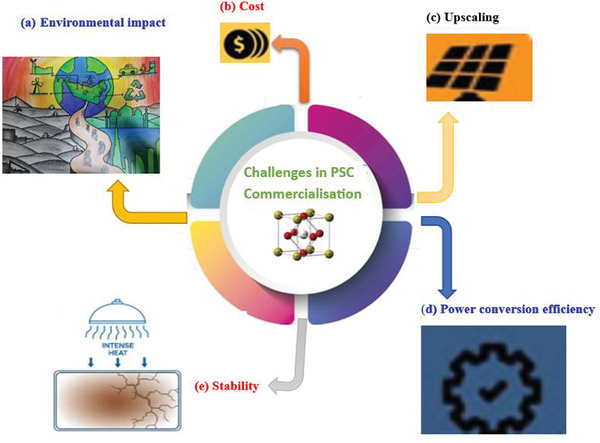
Industrial roadmap for perovskite commercialization Reproduced with permission [26]. Copyright 2024, Royal Society of Chemistry.

The continuous exploration of advanced materials and the subsequent introduction of PSCs to the market involve much more than achieving impressive efficiencies in the laboratory [12]. It requires balancing high performance with long‐term stability, scalable manufacturing, affordability, and environmental safety as summarised in Figure 3. Although PSCs have reached outstanding power conversion efficiencies (PCEs), maintaining this performance in large‐area modules and under real operating conditions remains a significant challenge [24]. Devices must withstand heat, moisture, and UV exposure for many years to be commercially viable. In addition, concerns related to lead content and environmental regulations must be addressed through effective encapsulation, recycling methods, and exploration of safer material alternatives. Alongside the growth in technical aspects, the greater interest lies in avoiding the life‐cycle impacts of PSC modules in evolving energy systems, including undesirable land‐use change, unnecessary resource extraction, and opportunities for low‐impact design [25]. Eventually, those who bear the greatest burden of serious environmental and health impacts associated with PSC technology deployment are residents near lead mining sites, and photovoltaic cell and PV module manufacturing sites [24]. Across the value chain, there is a need for enhanced stakeholder and citizen engagement to introduce equity and justice dimensions into low‐carbon transitions and to increase the social legitimacy of solar energy in communities affected by the undesirable impacts of PSC technology deployment [14]. Accordingly, strong collaboration among universities, industries, and governments is critical in transforming research advances into reliable, scalable, and sustainable solar technologies.

Just energy transitions to clean energy have become an increasingly urgent priority for policymakers, social scientists, and community activists alike [10]. Late‐stage consideration of the social impacts of PSC technologies results in inequities only after substantial effort, time, and money have been expended on research and development [27]. This is exemplified by concerns over the human health and environmental impacts of cobalt in lithium‐ion batteries [28, 29], the occupational hazards, land use, and consumption related to biofuels after decades of their existence [30, 31]. These lessons have shed light on the hurdles that may arise in just energy transitions to manage future research and decision‐making. Consequently, these concerns underscore the need to integrate justice considerations into early‐stage research and development. To this end, these discussions are often framed within the broader idea of a “just transition.” The materials and manufacturing, their implementation and disposal, the concept of energy justice, the compatibility of the SDGs, and the role of environmental microclimates should be considered throughout the technological lifecycle. Ongoing research undertakings have shown that early‐stage energy research is crucial in determining the design and impacts of PSC technologies needed for a clean energy future. The emerging innovative PSC projects will require new labour, land, and material resources to be cultivated for global production systems [21]. The infrastructure required by the technology is land‐ and metals‐intensive, shifting negative impacts on environmental groups, marginalized people, indigenous communities, endangered species, and farming lands [21]. In pursuit of transitions toward sustainable net‐zero carbon emissions, a deeper understanding is required on how injustice is embodied in energy production and supply chain systems [32]. It is possible to develop a justice‐weighted readiness framework for perovskite technologies that combines empirical performance data with socio‐ecological and ethical analysis. Therefore, PSCs are not appraised based on their efficiency in generating electricity, but on whether they impose environmental and health impacts on the real communities they are meant to serve [33]. As presented in Table 1, the most severe tension between solar industries and justice appears to manifest in land use and manufacturing phases. Whereas systematic assessment evaluates the technology's technical performance (power output, recycling efficiency, and lead emission rates) [34], life cycle assessments identify key concerns (contaminated soil and water systems, community exclusion, and exposure inequality) [35]. Taken together, these assessments provide an understanding of what is happening, who is affected, and whether the outcomes are fair, allowing the development of policies grounded in environmental safety and equity lenses.

**TABLE 1:** A practical framework that combines technical performance metrics with social justice considerations to evaluate whether PSCs can be deployed safely, ethically, and equitably across different environmental and community contexts.

Dimension	Systematic Assessment	Narrative (Justice‐Based) Assessment	Outcome for PSC Deployment	Refs.
Materials and device design	Lead content, efficiency, stability, encapsulation performance	Are safer designs prioritised for vulnerable communities?	Determines whether high‐risk materials are ethically acceptable	[36]
Environmental exposure	Lead leakage rates under heat, humidity, UV, and mechanical stress	Who is exposed to contamination (children, farmers, informal recyclers)?	Identifies risk inequality	[37]
Microclimate effects	Degradation under tropical, coastal, and arid conditions	Are hot and humid regions unfairly burdened with faster failure?	Highlights climate‐justice interactions	[38]
EoL management	Recycling efficiency, lead recovery, waste pathways	Are local recycling systems accessible and safe?	Evaluates circular‐economy justice	[39]
Energy access	Power output, low‐light performance, portability	Does PSC deployment reduce or reinforce energy inequality?	Links technology to SDG 7 and SDG 10	[40]
Agricultural integration	Agrivoltaic performance, greenhouse compatibility	Could lead to contaminated soil, food, and water?	Balances SDG 2 vs SDG 3 risks	[41]
Governance & policy	Existing regulations, standards, and compliance	Are communities included in decisions and protections?	Determines procedural justice	[42]

## 4 Previous Just Transition Case Studies on Energy Transition and Remediation Strategies

The idea of a just energy transition (JET) is about people, and has emerged from labour movements as a means to mitigate negative impacts on communities and workers in traditional energy production regions [43]. While the world urgently needs to move away from fossil fuels like coal, oil, and gas to address climate change, this shift cannot ignore the workers, families, and communities whose lives depend on those industries. A just transition recognises that climate action and social justice must go hand in hand. It asks a simple but powerful question: how do we clean up our energy system without leaving people behind? The concept did not start as a global climate policy idea. It grew from labor movements in the United States in the late 20th century [44]. Trade unions argued that when industries were shut down for environmental reasons, workers should not carry the burden alone. Over time, this worker‐centered approach expanded. It began to include broader concerns such as poverty reduction, community resilience, energy access, and fair economic development. By the time the Paris Agreement was adopted in 2015, the phrase “just transition” had entered international climate discussions, reflecting a global understanding that environmental progress must also be socially responsible [45].

History shows what happens when transitions are poorly managed. In parts of Europe and North America, coal‐mining regions experienced a rapid decline in the 20th century [46]. Mines closed, jobs disappeared, and entire communities struggled with unemployment and long‐term economic stagnation. These painful experiences taught policymakers that industrial change cannot simply be imposed from “above” – a position of authority. Without planning, consultation, and financial support, transitions can deepen inequality rather than solve problems. The concept of JET unfolds unevenly across sectors and geographies, with notable variations in procedural justice outcomes for communities and workers (cf. Table 2). An in‐depth analysis of JET policies from different countries across different development contexts shows that equitable outcomes require: (1) recognition justice (respect for local knowledge), (2) distributional justice (direct community benefit‐sharing), and (3) procedural justice (stakeholder participation) [47, 48, 49], as summarised in Table 2.

**TABLE 2:** Just energy transition case studies.

Country	Key Focus	Main Policy	Primary Solutions	Major Outcomes	Refs.
South Africa	Coal phase‐out	JET‐IP (∼$8.5B)	Decarbonisation + retraining and community planning	Progress has slowed, job concerns	[50, 51]
Indonesia	Coal reduction	JET‐IP (∼$20B)	Renewable investment and regulation reform	Finance gaps, slow disbursement	[52]
Vietnam	Coal and grid transition	JET‐IP (∼$15B)	Renewable growth + efficiency	Needs stronger stakeholder input	[53, 54]
Scotland	Oil and gas decline	National funds and skills programs	Job skills training and local investment	More equitable local benefits	[55]
Germany	Coal exit strategy	Coal exit commission	Worker rights and structural aid	Balanced policy with social guarantees	[56]

**Legend**: JET‐IP (Just Energy Transition Investment Plan)

Germany offers one of the typical examples of learning from past mistakes. As the country committed to phasing out coal, it did not simply announce plant closures. Instead, it created a coal commission that brought together government officials, industry leaders, trade unions, scientists, and civil society representatives [57]. The aim was to design a transition roadmap that balanced environmental goals with economic security. Large financial packages were set aside to support affected regions, fund infrastructure projects, and retrain workers. By investing in regional development and ensuring worker compensation, Germany sought to make the transition structured and predictable rather than sudden and disruptive [58]. In the United Kingdom, particularly in Scotland, the decline of North Sea oil and gas has prompted similar thinking. Policymakers recognised that renewable energy, especially offshore wind, could offer new opportunities, but only if workers were supported in moving between sectors. Training programs, transition funds, and regional diversification strategies were introduced to ease this shift. Although challenges remain, especially in ensuring comparable wages and job security, the effort reflects a more human‐centred approach to energy policy. Despite the ambitious global efforts to JET, equitable outcomes have been persistently undermined by implementation barriers [59]. As depicted in Table 3, the cross‐cutting challenges observed in transitioning economies are not limited to system inequalities, stakeholder exclusion, slow deployment, and funding gaps. These barriers manifest in different scales and thus require integrated solutions since policy ambition cannot overcome system barriers.

**TABLE 3:** Common challenges in just energy transitions.

Challenge	Description
Funding Gaps	Pledged climate finance often falls short of actual investment needs [60].
Stakeholder Inclusion	Local communities and workers are sometimes excluded from planning and benefits [60].
Slow Implementation	Renewable deployment and coal retirement timing lag behind targets [61, 62].
Systemic Inequities	Structural inequalities make equitable benefit sharing harder to achieve [63, 64].

In developing countries, just transition takes on additional complexity because economic development and poverty reduction remain urgent priorities [65]. For instance, in South Africa, the electricity system relies heavily on coal, which provides employment to thousands of people and supports entire local economies [66]. Rapidly closing coal plants without alternative jobs would risk severe social consequences. To address this, South Africa developed a JET Investment Plan with financial support from international partners. The plan combines renewable energy development with worker retraining, community engagement, and regional economic diversification. While implementation has been slower due to funding and structural challenges, the framework demonstrates how international cooperation can help balance climate goals with social protection. Similarly, Indonesia and Vietnam depend significantly on coal to power industrial growth. Through JET partnerships, they have sought international financial support to retire coal plants early, modernise their grids, and expand renewable energy. However, these efforts also reveal the complexity of balancing economic growth, energy security, and climate commitments. Funding delays, regulatory reforms, and coordination challenges have slowed progress. Still, the inclusion of social considerations in national energy planning marks an important shift from earlier, purely technical approaches to energy policy.

A burgeoning body of literature underscores the need for people to be involved in shaping the transition to clean energy [67, 68, 69]. When workers, communities, and local leaders participate in decision‐making, policies are more likely to gain public trust and long‐term support. Moreover, in order to deliver justice, reliable financing is essential through real investment in new industries, infrastructure, and education. Energy justice should also be implemented through re‐training and skills development [70]. Without clear pathways to new employment, displaced workers face uncertainty and hardship. Generally, regional economic diversification ensures that communities do not replace one dependency with another. In the past decades, industrial shifts often prioritised economic efficiency over social well‐being, leaving communities to deal with the consequences [71]. Today's transition frameworks should seek to combine climate ambition with fairness, recognising that sustainable development must include social stability, decent work, and inclusive growth [72, 73]. Yet the growing global emphasis on fairness in climate action reflects a deeper understanding: transitioning to clean energy is not only an environmental necessity, but also an opportunity to build more resilient, equitable societies [73]. When designed thoughtfully, the shift away from fossil fuels can create new jobs, strengthen communities, and ensure that progress toward a low‐carbon future benefits everyone, not just a few.

Vulnerable populations are forced to bargain over what could be termed a temporal and spatial dual bind [74]. These spread the immediate, local costs of energy transition infrastructure, either in the mining of key mineral resources, or construction of transmission lines or land repurposing, and the global returns of decarbonisation, climate stability, and emission reduction, are so distant, both spatially and temporally. This dynamic constitutes a space of continuous sacrifice, in which individuals are instrumentalised to secure benefits they may never receive directly. As a result, the rapid pace of technological adoption does not align with the sluggish pace of cultural ecology. This leaves people to go along with changes rather than react to them, uprooting customary ways of life.

Even noble policies regarding renewable energy can perpetuate structural inequalities [75]. Large‐scale projects that are attractive to big businesses are habitually preferred to decentralised community‐based solutions that are not adequately funded. There are also financial and technical barriers that lock the uneducated or low‐income communities out. Shifts from decentralised coal/biomass to centralised electric grids require new social institutions (regulatory agencies) and hierarchical labour hierarchies (unskilled operators versus skilled technicians). Therefore, these co‐evolutions indicate that future energy transitions including PSC module will likewise reshape social and labour organisations with outcomes dependent on both technology and governance choices [76]. Political power can be dismantled through the de‐platforming of collective labour, the shift to geographically decentralised renewable infrastructure, the redistribution of power, and the destruction of formerly formed solidarity. It could mean confronting corporate and state forces on their own terms and interfering with the established forms of resistance and negotiation in rural or indigenous communities.

Furthermore, low‐income economies are rhetorically required to ‘adapt’ to energy transition and climate change [77]. This expectation masks a structural injustice: vulnerable economies must absorb adaptation costs and implement climate policies, while wealthy nations, responsible for emissions, retain decision‐making power over transition terms. Accordingly, this represents adaptive coercion, in which vulnerable populations are forced to bear the burden of change while being excluded from choices that shape outcomes [78]. Resilience rhetoric masks coercion –communities to externally accept externally‐imposed systems while obscuring their loss of autonomy, the opposite of actual autonomy. Here, politics of recognition and restructuring the system is required to respond to these preconditions of weakness [78]. Besides the conventional stakeholder consultation, the energy transitions ought to be mindful of the law and institutions that recognise the rights of nature, non‐human entities, and the global relationships that communities safeguard. The affected populations should be given real decision‐making powers by the decision‐making structures, such as veto powers, over their land and resource optimisation [79].

## 5 The Role of Perovskite Solar Cells in the Green‐Energy Transition

PSCs are among the most notable technologies in photovoltaic research since silicon cells were first reported [12]. In practice, lead‐based perovskite films can convert sunlight into electricity with much higher efficiency and compete with the most mature silicon technology [15]. Perovskites can be fabricated by solution processing at low temperatures, in contrast to silicon, which requires high temperatures, costly vacuum systems, and complex crystal‐growth procedures [34, 35, 36], and can be printed and coated in the same way as the graphics and packaging industries. This significantly reduces thermal requirements, capital expenditure, and technical challenges in building solar cells [80]. In principle, roll‐to‐roll production can enable large‐scale fabrication on flexible substrates such as plastic, metal foils, and glass, significantly lowering production costs [81]. From the resource perspective, lead is relatively abundant and found in numerous locations around the world, and thus offers significantly fewer supply‐chain vulnerabilities than some other materials competing in thin‐film applications that require rare or scarce elements [82]. It is more cost‐effective to use lead‐based absorbers in perovskite solar cells because the raw materials for this technology are more consistent and cheaper than those for CIGS or CdTe. Economically, the aforementioned features lead to a future in which solar energy will be among the least expensive sources of energy ever utilised. Compared to the traditional silicon‐based panels, PSCs are therefore ideal for decentralised energy access in vulnerable and infrastructure‐limited settings [80, 83]. It is widely projected that perovskite modules will cost less than $0.20 per watt once manufacturing processes mature. Besides, it can provide a wide ranging economical and social benefits when deployed inclusively. Evidence from prior studies links successful solar energy deployments to improved human development, job creation, and poverty reduction [84]. Similar reports have shown that off‐grid solar installations in sub‐Saharan Africa have improved educational outcomes and household income by enabling lighting for study and productivity after dark [85, 86, 87]. Conversely, in health care settings, renewable energy supports the operation of medical devices and the refrigeration of vaccines in remote clinics [88].

From a sustainability perspective, chemical measures have been developed that convert lead into insoluble, stable compounds upon exposure, thereby preventing its entry into soil or water systems [12]. The technology is also being developed alongside recycling procedures so that almost all of the lead will be recycled and re‐used at the end of the module's lifetime, rather than being released to the environment [24]. With all these protections in place, the actual threat of lead leakage into the environment will be minimal compared to the potential harm of continuous usage of coal, oil, and gas in public health and the environment [24]. Notably, PSCs have tunable band gaps, enabling engineers to create cells optimised for sunlight, indoor lighting, or use with silicon. This makes them suitable for powering both small sensors and Internet‐of‐Things (IoT) devices, as well as towers and data centers [89, 90]. Specifically, perovskite‐silicon tandem modules can enable upgrading existing photovoltaic infrastructure to increase energy production on the same footprint without necessarily overturning the entire manufacturing ecosystem [15]. The lead‐based PSCs can presently operate for thousands of hours at elevated temperatures, in high humidity, and under constant light, and is approaching commercial durability requirements through chemical and structural modifications [24]. The studies of two‐dimensional and mixed‐dimensional perovskite structures, grain‐boundary passivation, and lead‐sequestration layers are advancing stability, which underlines the argument for practical implementation [24, 91]. Though lead‐free devices, including those based on tin are currently under investigation, they are yet to achieve the efficiency, stability and reliability of lead‐based devices [92, 93]. They are prone to rapid oxidation, deep defects and unpredictable electronic behaviour in most instances, and are associated in other cases with their own toxicity problems. It is projected that lead‐based PSCs will be the most technologically feasible for realising high‐performance, low‐cost, and scalable perovskite photovoltaics [24, 94].

### 5.1 Community Solar Gardens: Expanding Access to Renewable Energy for Low‐Income Communities

**FIGURE 4:**
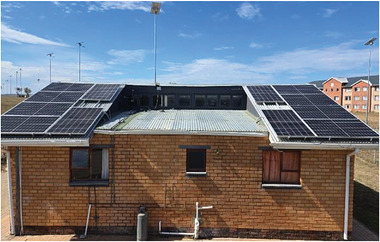
Community solar gardens: expanding access to renewable energy for low‐income communities.

Improving the affordability and accessibility of distributed generation (DG) solar technologies is essential for achieving an inclusive and sustainable energy transition [95]. The expansion of DG solar can be attributed to its multiple benefits, including reducing reliance on traditional electricity sources, lowering consumer energy costs, and mitigating climate change [96]. The increasing adoption of DG solar has also been driven by declining installation costs and supportive governmental policies that encourage the use of renewable energy. Community solar gardens (CSGs) have demonstrated that renewable energy transitions can serve low‐ and moderate‐income (LMI) households equitably [97] as depicted in Figure 4. CSGs have been identified as energy‐access tools for energy‐insecure but creditworthy populations. CSGs require attention in subsidy redistribution, ownership democratisation, and regulatory reforms since technology alone cannot guarantee energy justice. Studies have shown that trusted community partners, such as local non‐profits, faith‐based organisations, and community action agencies, play a critical role because they have established credibility and social connections [98]. Unlike higher‐income groups who may respond well to social media or online campaigns, LMI communities are more responsive to direct outreach, including door‐to‐door visits, utility mailings, and personal phone calls. Community partnerships are more effective through training and technical support, community control over program design, and adequate funding for sustained engagement [99].

Physical presence in the community through local events, meetings, or flyers posted in public spaces like libraries and grocery stores is also highly effective, allowing organisers to meet residents where they are. This approach addresses key barriers the households face, including limited internet access, language differences, and distrust stemming from past experiences with predatory energy offers. Peer‐to‐peer networks further reinforce credibility, showing residents that real financial savings from solar are achievable [99]. Additionally, incorporating multi‐lingual materials and non‐digital communication channels ensures that all community members, including non‐English speakers, can access and understand the program. Overall, Figure 4 serves as a roadmap for inclusive and effective engagement, demonstrating that building trust and using high‐touch, community‐centred communication strategies are essential to expanding renewable energy access to those who stand to benefit most economically and environmentally.

One common policy that promotes DG solar is net metering. Net metering allows DG solar customers to purchase electricity from the grid when needed and to sell any surplus electricity generated back to the grid [100]. While this policy encourages renewable energy use, it can also create “stranded costs” for utility providers due to decreased demand for conventional electricity. Utilities may attempt to recover these costs by increasing rates, which can inadvertently raise energy bills for non‐DG solar customers. These cost shifts tend to disproportionately affect low‐income households, which often face significant financial and structural barriers to accessing DG solar. Table 4 presents the features of community solar gardens.

**TABLE 4:** Overview of how community solar gardens improve access to affordable distributed solar energy while promoting energy equity in low‐income communities.

Aspect	Description	Relevance to Low‐Income Communities	Refs.
Community solar gardens (CSGs)	Shared solar photovoltaic systems, where multiple households subscribe to or benefit from a single solar installation	Enables access to solar energy without the need for rooftop ownership or high upfront investment	[101]
Distributed generation (DG) Solar	Small‐scale, decentralised solar energy systems located close to the point of consumption	Reduces dependence on centralised fossil‐fuel‐based electricity and enhances energy resilience	[102]
Affordability	Lower upfront costs through shared ownership or subscription‐based models	Makes solar energy financially accessible to households with limited capital	[103]
Energy cost reduction	Solar‐generated electricity offsets grid electricity consumption	Leads to reduced monthly electricity bills and improved household energy security	[104]
Declining installation costs	Technological advancements and economies of scale have reduced solar PV costs.	Lowers participation barriers for community‐based solar projects	[105]
Government policy support	Incentives such as tax credits, subsidies, and renewable energy mandates	Encourages investment in community solar projects targeted at underserved populations	[106]
Environmental benefits	Reduced greenhouse gas emissions and fossil fuel reliance	Supports climate change mitigation while improving local air quality	[107]
Social equity	Inclusive energy access through shared renewable infrastructure	Addresses energy poverty and promotes equitable energy transitions	[108]
Grid benefits	Reduced transmission losses and improved grid stability	Enhances the reliability of the electricity supply in vulnerable communities	[109]

Two major challenges are the upfront installation costs and the prevalence of rental housing among low‐income populations. Many low‐income individuals live in multi‐family buildings where they do not control the roof space, limiting their ability to install solar panels even if they could afford them [110]. As a result, these households are often excluded from the financial and environmental benefits of DG solar. CSGs offer a potential solution by providing shared solar resources that multiple households can access, including renters and those who cannot afford individual installations [111]. By allowing low‐income communities to participate in solar energy generation without requiring direct installation, community solar programs can promote equitable access to renewable energy while helping to reduce overall energy costs.

### 5.2 Deeper Dimensions and Artificial Intelligence (AI) in Energy Transitions

**FIGURE 5:**
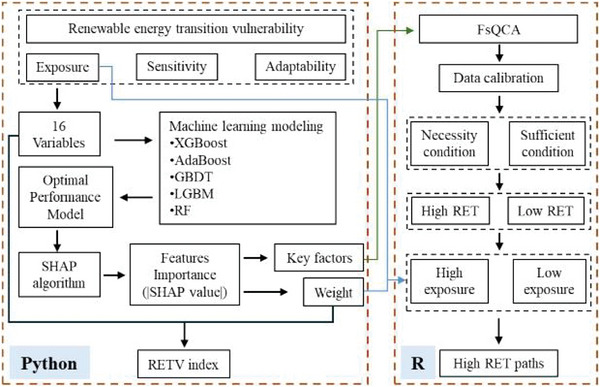
Deeper dimensions of vulnerability in energy transitions. Reproduced with permission [115]. Copyright 2026, Elsevier.

Transitions in energy is characterised in a singular techno‐scientific sense [112], with a focus on productivity and rationality in the reality of communities whose world‐vision is relational. Research shows that many rural and indigenous communities, mountains, rivers, and land embody cultural significance beyond resource extraction [113]. They function as common sites for cultural practice and social identity. Large‐scale renewable energy projects may disrupt these ecological‐cultural relationships by altering habitats and displacing natural resources [113]. This ecologically grounded knowledge system of land stewardship and sustainable land use that these communities have been building over generations is generally overlooked [114]. This is a kind of epistemic injustice that undervalues place‐based intelligence, which is vital to sustainable energy design. Figure 5 presents a detailed methodological framework for evaluating the complex dimensions of vulnerability in renewable energy transitions (RET) through a dual computational approach combining Python and R, where R stands for the first letters of the names of its two creators: Ross–Ihaka and Robert–Gentleman.

The process starts by defining Renewable Energy Transition Vulnerability (RETV) in terms of exposure, sensitivity, and adaptability, operationalised via 16 key variables [116]. In the Python phase, machine learning algorithms such as XGBoost, AdaBoost, GBDT, LGBM, and Random Forest are used to identify the best‐performing model [117]. The results are then interpreted using the SHAP (Shapley Additive exPlanations) method, which quantifies feature importance and assigns weights to calculate the final RETV index. Simultaneously, the framework moves into the R environment to conduct Fuzzy‐set Qualitative Comparative Analysis (fsQCA) [118]. Here, the data is calibrated, and necessity and sufficiency conditions are examined to uncover causal combinations that lead to high or low RET and exposure levels. By integrating quantitative machine learning insights with qualitative comparative analysis, this dual‐pathway approach highlights the key drivers of vulnerability and resilience, revealing the “high RET paths” that shape how regions navigate the transition toward sustainable energy.

Within the context of the clean energy transition, artificial intelligence (AI) has emerged as a compelling driver of innovation, offering overall insights on the overall feasibility of low‐carbon energy systems [19]. Besides the technical enhancements, AI may provide a prototype of how energy may be produced, distributed, and made accessible to the human population [119]. AI addresses many of these complexities by detecting real‐time shifts in supply and demand, guiding the optimal dispatch of renewable resources [19]. The most influential aspects of AI implementation are predictive analytics and energy forecasting. Renewable sources of energy, particularly solar and wind, are intermittent and variable, posing a challenge to grid stability [120]. Smart AI‐driven systems based on machine learning and deep learning can produce forecasts of energy production and demand with very high precision, using past weather patterns, current sensor readings, and historical usage patterns. With this predictive capacity, grid operators can balance supply and demand and achieve the maximum integration of renewable energy. In PVs, AI can be used to track module performance and predict solar irradiance, delivering maximum energy and reducing operation time [121].

**FIGURE 6:**
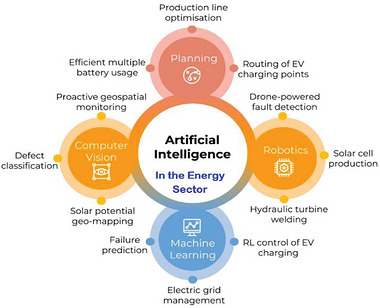
Artificial intelligence in the clean energy transition. Reproduced with permission [125]. Copyright 2026, Elsevier.

As shown in Figure 6, AI is no longer just a supporting technology; it is becoming a central engine of the global clean energy transition. By integrating tools such as machine learning, computer vision, and robotics, AI is transforming power generation and grid distribution to end‐use consumption [122]. Machine learning acts as the analytical core of modern energy systems, enabling accurate demand forecasting, smarter grid coordination, and improved reliability by predicting equipment failures before they happen. This is particularly important for stabilising smart grids that rely on variable renewable energy sources such as solar and wind [123]. Concurrently, computer vision enhances monitoring and diagnostics by identifying optimal solar installation sites and detecting defects in PV modules, such as microcracks and hotspots [124].

Robotics further enhances performance and safety, with autonomous systems cleaning solar panels in dusty regions and drones or climbing robots inspecting wind turbines without exposing workers to risk. Beyond operations, AI also supports strategic transition planning by optimising renewable manufacturing processes, improving load balancing in electric vehicle charging networks to manage peak demand challenges such as the “duck curve” [126]. It also plays a critical role in accelerating the discovery of advanced materials for next‐generation batteries and electrolysers. From policy design to on‐the‐ground implementation, AI is helping shape a cleaner, more resilient, and economically sustainable global energy system capable of meeting long‐term climate action goals [127].

AI has also emerged as a powerful catalyst in controlling of intelligent grids and decentralised energy grids, such as microgrids, in remote or underserved locations [19]. An intelligent algorithm can dynamically allocate energy resources to distributed systems, manage energy storage, and project local demand. With AI, energy waste will be reduced, equitable energy access will be enhanced as battery consumption and load allocation will be optimised [119]. Another reason renewable energy systems are more efficient in operation is predictive maintenance. AI will be able to identify the state of equipment, address potential errors in PV cells or wind turbines, and recommend preventive actions [128]. This enhances the life of the energy infrastructure and reduce downtime and repair costs. In more advanced technologies, e.g., lead‐based PSCs, AI may be applied to monitor environmental stressors and degradation, and allowing safer recycling strategies. Besides technical optimisation, AI also influences strategic energy planning and policy development. AI can enable policymakers to make trade‐offs, as energy systems are complex and can be modelled to simulate various deployment scenarios to understand their social and environmental impacts [129]. This is particularly relevant in the context of emerging economies, where the demand for greater energy access should be weighed against environmental sustainability and social equity. AI can be able to identify areas with a high probability of renewable energy, forecast future demand, and recommend implementation strategies that are effective, accessible, inclusive and equitable [19].

AI application is also significant to energy affordability. Residential and commercial users can also use AI to lower energy costs and ensure an uninterrupted electricity supply by enabling dynamic pricing, load optimisation, and demand management [130]. In community‐based energy or decentralised energy systems, AI can be employed to ensure that energy resources are effectively allocated and that needy services are given priority. Furthermore, the capability of AI can be leveraged in storage and dispatch optimisation to make clean energy cheaper and more affordable for low‐income citizens [131]. Also, AI allows fostering the development of sustainability and environmental stewardship within energy systems. It will be in a position to trace the impacts on the environment, track reductions in emissions, and reinforce the recycling and management of resources used in PV technologies. By linking operational performance to the environment and social responsibility, AI is a tool that enables transition to clean energy, which is both technologically, ecologically, and socially oriented. In view of this, AI is one of the fundamental drivers of a successful, effective, and equitable clean energy transition [19]. Smart grids enable AI to enhance the functionality and accessibility of renewable energy systems such as predictive analytics, smart grid management, decentralised energy optimisation, anticipatory maintenance, and policy modelling [132]. The application of AI can ensure that the new solar PV, wind, and PSC technologies are turned into reliable, and sustainable energy for all people, bridge the energy access gap, and promote environmental and socio‐economic priorities [133].

## 6 Perovskite‐Powered DC Microgrids: Bridging Efficiency, Resilience, and Energy Justice in Smart Cities

It is worth noting that direct current (DC) microgrids are increasingly seen as a paradigm shift in smart‐grid architectures designed to address energy sustainability, and equity challenges of rapidly urbanising smart cities [131]. Unlike the traditional centralised alternating current (AC)‐based power systems, DC microgrids utilise localised networks that directly incorporate distributed renewable energy sources, energy storage systems, and DC loads [134]. This architecture simultaneously minimises power conversion losses and the system's efficiency and reliability, especially in urban areas where photovoltaic generation, battery storage, electric vehicles, and digital infrastructure largely use DC power. Due to this diverse array of applications, DC microgrids are envisaged to offer a more viable and technologically feasible direction for contemporary urban energy systems.

**FIGURE 7:**
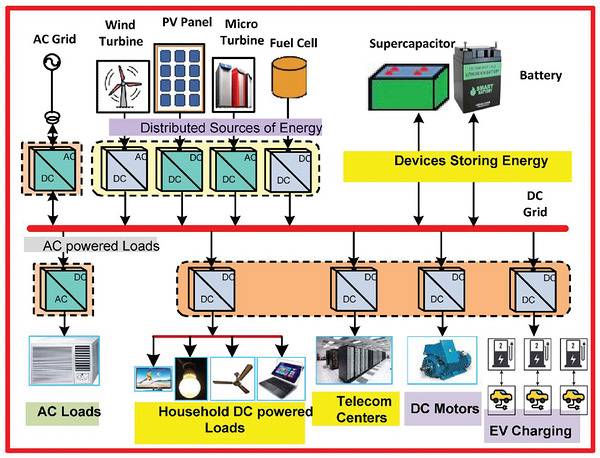
Perovskite‐powered DC microgrids: bridging efficiency, resilience, and energy justice in smart cities. Adapted with permission [140]. Used under CC BY 4.0, MDPI.

The DC microgrid architecture integrates diverse renewable energy sources, including PV panels [135], wind turbines [136], microturbines, and fuel cells, as well as batteries and supercapacitors [137], to supply both AC and DC loads directly, as shown in Figure 7. In the context of Perovskite‐Powered DC Microgrids, PSC can serve as a high‐efficiency PV component, boosting overall energy conversion efficiency while enabling compact, scalable deployment in urban environments. By directly connecting distributed energy sources to DC‐powered household loads, telecom centres, DC motors, and electric vehicle (EV) charging stations, the system minimises AC‐DC conversion losses, enhancing resilience and reliability [135]. Moreover, integrating perovskite PV with local storage and intelligent load management supports energy justice, ensuring equitable access to clean and affordable power in smart cities while enabling seamless integration with the broader AC grid for stability [138]. This harmonised approach demonstrates how advanced materials and smart microgrid design can simultaneously address efficiency, resilience, and socio‐technical equity in urban energy systems.

Microgrids led by community members and decentralised systems must be prioritised for financial and technical assistance, not as pilot projects but as the primary elements of a fair energy future. Nonetheless, the shift to renewable energy will not only help avoid the repetition of past hierarchies and exclusions but also enhance equitable distribution of energy resources. Still, it will also be defined with the consent of communities, in terms of their sets of values, knowledge, and worlds of relations. Equitable energy transitions require transformation across the socio‐technical system [139]. Transitions that optimise PSC efficiency while centralising external toxicity costs, corporate control or imposing systems without community consent represent technical change without equitable transformation.

In the broader context of a fair transition to clean energy access, DC microgrids play a vital facilitative role through decentralised energy production and inclusive access to electricity [141]. Their scalability and modularity can be applied across a wide range of urban environments, thereby reducing energy poverty and enhancing local energy resilience. DC microgrids can facilitate community‐scale ownership models and local energy governance by fostering job creation, environmental responsibility, and social equity, among other benefits [142]. The emerging PSCs technology also enhances the capabilities of DC microgrids, owing to their superior optoelectronic properties and their ability to integrate with DC systems [143]. PSCs can be more efficient than most conventional photovoltaic technologies, delivering high efficiencies and low‐temperature processing, making them attractive for building‐integrated photovoltaic applications [12]. These features are especially beneficial in intelligent cities, where size limitations and built‐in aesthetics are important factors. Besides, the DC output of PSCs enables direct connection to DC microgrids, reducing the need for inverters and enhancing overall system efficiency.

Notably, renewable energy will play an important role in enhancing social equity and inclusive economic development by increasing access to reliable energy for underserved communities especially in remote areas and peri‐urban settings [144]. Off‐grid solar systems and mini‐grids can deliver electricity to rural and low‐income communities, thereby improving healthcare, education, and economic activities [145]. Growth of the renewable energy sector also provides job and entrepreneurial opportunities at the local level, promoting skills development and income generation. Moreover, in the long run, renewable energy reduces energy costs and dependence on imported fossil fuels, increasing economic stability and making development gains more equitable [146]. A sustainable and inclusive energy transition guided by the principles of a just transition aims to meet climate targets and protect social and economic fairness [147]. It entails providing workers and communities that depend on fossil fuel industries with skills training, employment, and sufficient social safety measures as energy systems transition to cleaner energy sources. Concurrently, it facilitates fair access to affordable, reliable, and clean energy through inclusive planning and community‐based solutions. Good governance, extensive stakeholder participation, and equitable investment allocation are key to ensuring that the gains and burdens of the transition are shared, ensuring social justice and long‐term economic and environmental sustainability [148]. Another critical prospect is integrating lead‐based PSCs into DC microgrids to make clean energy more accessible at a lower cost. It has the potential to reduce capital expenditure by simplifying balance‐of‐system components and reducing material consumption in perovskite‐based DC systems. Therefore, decentralising renewable energy to be accessible to underserved populations [149]. Also, the potential application of PSCs in indoor and low‐light conditions is beneficial for future smart‐city applications, such as the Internet of Things (IoT), sensors, and smart building systems.

**FIGURE 8:**
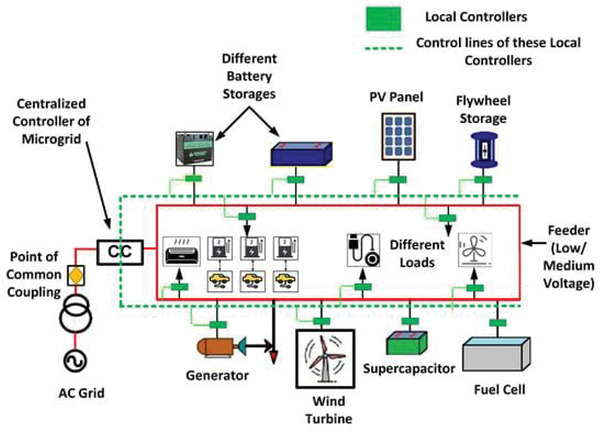
Fundamental configuration of a DC microgrid. Adapted with permission [140]. Used under CC BY 4.0, MDPI.

DC microgrids that combine PSCs based on lead and other advanced energy materials represent a potential convergence of smart‐grid design and just‐transition principles [150]. This paradigm facilitates the creation of inclusive, low‐carbon smart cities by enabling them to adopt efficient, resilient, and decentralised power systems. With the right policy support, technological protection, and community involvement, DC microgrids powered by PSC technology can be central to advancing clean energy access [151]. Further, these microgrids will address the needs of urban energy transitions, thereby establishing a broader social and environmental agenda. Figure 8 presents the basic architecture of a DC microgrid. In this setup, different renewable energy sources – photovoltaic panels and wind turbines are combined with energy storage technologies, including flywheels and super‐capacitors. All these units are linked to a shared low‐ or medium‐voltage DC bus, where local controllers regulate power conversion and deliver electricity to residential or industrial loads. At the system level, a centralised controller (CC) supervises the entire network. It coordinates the local controllers to ensure smooth power flow, maintain voltage stability, and optimise overall performance [135]. The microgrid is also connected to the main alternating current (AC) grid through the point of common coupling (PCC), allowing controlled energy exchange. This configuration improves energy efficiency and reliability by enabling flexible operation. The microgrid can function in grid‐connected mode under normal conditions or switch to an autonomous “island” mode during outages, ensuring a continuous power supply. The microgrid provides various DC and AC loads and can communicate with the main AC grid via the PCC [140]. This is a centralised controller, in combination with local controllers and communication links, that provides overall control and ensures effective power control and stable operation across low‐ and medium‐voltage feeders.

### 6.1 Lead Degradation and Environmental Justice in PSC Deployment

Despite their high efficiency and cost‐effective fabrication methods, lead‐based perovskite solar cells (PSCs) face significant barriers to commercialisation and widespread exploitation due to lead toxicity and vulnerability to degradation [16, 93]. While efforts have been made in recent years to develop lead‐free perovskite variants, and attempts to reduce lead leakage from PSCs to mitigate environmental and public health risks, such strategies are still at an early stage [12, 152]. A degradation study is essential for assessing the reliability and practicality of lead‐based PSCs intended for large‐scale use in clean energy applications. Despite their remarkable efficiencies, these materials have short operational lifetimes because they are sensitive to moisture, oxygen ingress, heat, and ultraviolet radiation [153]. Such environmental pressures cause chemical disintegration of the perovskite lattice leading to ion migration, and reduced chemical stability which are detrimental to device performance. Moreover, interfaces between the perovskite absorber and the charge‐transport layers degrade, leading to the development of defect states and adverse interfacial reactions that degrade device performance [12]. An in‐depth understanding of these processes is thus required to enhance device encapsulation, material structure, and interface engineering, making solar modules more stable and durable.

**FIGURE 9:**
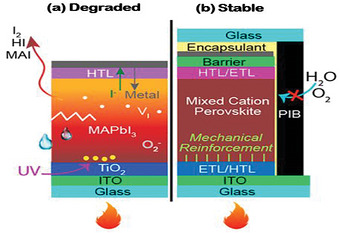
Degradation analysis of lead‐based perovskite solar cells [155].

With reference to Figure 9, the degradation analysis clearly shows that lead‐based perovskites, such as methylammonium lead iodide (MAPbI_3_), are particularly vulnerable to environmental stressors [24]. Environmental stressors weaken the crystal framework, ultimately leading to structural instability and loss of performance. However, a practical pathway toward improved durability and immobilising lead leaching includes the use of encapsulants [135]. By introducing mechanical reinforcement strategies and applying strong encapsulation layers such as polyisobutylene (PIB) barrier films, researchers can effectively shield the device from external contaminants [154]. These protective measures significantly enhance the stability of mixed‐cation perovskites and ensure more reliable long‐term device performance.

In the context of a just transition, the degradation analysis is also very important for determining environmental safety and social risk in lead‐containing PSCs [91]. Damaged and/or decommissioned devices may reveal the perovskite layer due to mechanical damage, moisture ingress, or encapsulation failure, leading to the leaching of lead compounds into nearby ecosystems [156]. This danger is especially acute in low‐income or rural areas, where waste management and recycling infrastructure can be insufficient. Degradation analysis helps in developing effective protective measures, including lead‐sequestration layers, improved encapsulation materials, and circular recycling plans by measuring the impact of degradation on lead release and device failure rates [91]. Through this, degradation analysis will be used to ensure that lead‐based PSCs can contribute to access to clean energy without imposing new environmental or public‐health costs, and to aid a more equitable and responsible energy transition.

### 6.2 Environmental Impact of Photovoltaics

Photovoltaic (PV) technologies have become one of the pillars of the global effort to decarbonise energy systems. These innovative technologies are fundamental to reducing greenhouse gas emissions and addressing the increasing global energy demand [157]. PVs are not ecologically or socially free, despite their significant environmental advantages compared to fossil fuels. These effects cannot be fully comprehended in a detached way, and there must be a comprehensive approach that would make the shift to solar energy sustainable and fair. The environmental impact of PVs starts with their manufacturing. The production techniques for silicon‐based and thin‐film modules entail energy‐intensive processes and the use of various metals and chemicals, including silver, aluminium, and cadmium [158]. Such processes can lead to greenhouse gas emissions, chemical effluents, and particulate pollution, requiring responsible production practices. Big PV systems also require substantial land, which may disrupt local ecosystems, fragment habitats, and alter soil and water processes [159]. Though these impacts are typically less severe than those from extracting fossil fuels, they require greater attention of site selection and mitigation strategies to safeguard biodiversity and ecosystem services.

**FIGURE 10:**
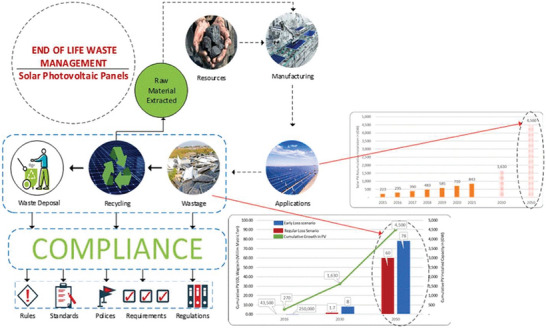
Environmental policy, law, and responsibility in photovoltaic technologies. Reproduced with permission [163]. Copyright 2026, Elsevier.

Emerging PV technologies, particularly PSCs have other environmental implications since many of their high‐efficiency devices contain lead [160]. The likelihood of release when degrading modules, breaking them accidentally, or discarding them at the end of life may pollute soil and water systems. Some important strategies for reducing these risks include encapsulation, recycling programs, and the development of low‐lead or lead‐free perovskite versions [24]. The PV life cycle needs to be regulated and monitored to ensure safe handling, transportation, and disposal of hazardous materials. Figure 10 illustrates the full life cycle of PV panels, showing that clean energy does not end at installation but extends to responsible EoL management. As global solar capacity is projected to reach about 4500 GW by 2050, the world will also face a significant rise in discarded panels. The projections are 60–78 million metric tons of waste from both early replacements and panels, reaching their 25–30‐year lifespan [161]. The framework emphasises the need to shift from a simple “use and dispose” model to a circular approach that recovers valuable materials through recycling. In this pursuit, strong regulatory compliance through standards, policies, and enforceable laws ensures accountability and supports sustainable lifecycle management [162]. Ultimately, the sustainability of solar energy depends not only on clean power generation but also on how responsibly we manage its materials at the end of their life (cf. Figure 10).

In addition to issues related to chemicals and materials, PV deployment also has significant interactions with the social environment. Major projects have the potential to influence land access, livelihoods of the local population, and even cultural landscapes [164]. In contrast, small‐scale rooftop or community systems can impact local energy governance and equity [165]. Planning in a socially sensitive manner entails involving communities in the decision‐making process, ensuring equitable distribution of benefits, and considering the unintended consequences of such as a lack of access to energy resources. This is consistent with the general concept of environmental justice, which holds that vulnerable populations must not be subjected to unequal environmental impacts and must not be prevented from enjoying the benefits created by renewable energy [73]. One way through which policies and programs should work is therefore to encourage inclusive participation, community‐based energy programs, and equitable access to energy and economic gains. The core of reducing the adverse effects of PV systems lies in environmental management practices. They should source materials, implement chemical and waste management practices, ensure ecological oversight, and implement robust end‐of‐life recycling programs to help reduce direct and indirect environmental impacts [166]. Life‐cycle assessments can inform decision‐makers about the hotspots of environmental impacts, both at the resource extraction level and at the module disposal level, and ensure that interventions focus on the areas of greatest concern.

Reckless PV deployment is assessed using environmental impact assessments (EIAs) as a pre‐requisite [167]. By assessing potential ecological, social, and health impacts before project approval, EIAs provide a structured framework for mitigation, monitoring, and community engagement. The introduction of the long‐term factor, which includes cumulative environmental impact, material toxicity, and socio‐economic impact, is sufficient to ensure that PV projects do not cause unintended harm. PV technologies are a low‐carbon alternative to traditional energy, with environmental implications at the ecological, chemical, and social levels [168]. The only means of ensuring a sustainable and fair energy transition is to consider the environment and to assess and proactively involve impacted communities, especially in cases involving high‐risk materials such as lead. It is only through this type of complex interaction that solar energy can become what it can be, by promoting equitable development and the decarbonisation of the energy sector.

## 7 Environmental Policy, Law, and Responsibility in Photovoltaic Technologies

The adoption of PV technologies, e.g., the creation of lead‐based PSCs, must be closely connected with ecological policy, regulatory frameworks, and proactive responsibility to ensure that sustainability goals are achieved [91]. Despite PVs being widely commended for their capacity to reduce greenhouse gas emissions, their production, use, and disposal procedures pose environmental and occupational issues that must be managed. Occupational health and safety are one of the main concerns in PV manufacturing [25]. The solvents, metals, and chemicals most commonly encountered by workers including lead and cadmium, are toxic [25]. The policies and regulations require implementing comprehensive safety measures such as wearing personal protective equipment, maintaining controlled ventilation, taking precautions when handling chemicals, and conducting regular inspections. The enforcement of such standards will ensure that workers do not experience inhalation or dermal exposure, minimise accidents, and that PV manufacturing is consistent with broader ethical and environmental responsibilities [65].

Lead is a major component of high‐efficiency perovskite solar cells and is considered an additional challenge [63]. The process of sourcing raw lead compounds and cultivating them through to fabrication may pose a major threat to workers, the communities surrounding it, and the environment. The strict measures in environmental legislation encompass storage, transportation, and production, such as secure storage and handling using closed‐loop systems and specialised employee training. These measures ensure that lead exposure is reduced and accidental discharges are controlled as quickly as possible, thereby reducing the risk of long‐term ecological contamination [12]. Another important point to note is how PV modules are to be handled at the EoL. The recycling policies and programs will be necessary to recover precious metals, neutralise hazardous materials, and prevent environmental pollution. Materials such as silver, gold, and perovskite can be re‐used through take‐back programs, chemical recycling complexes, and closed‐loop recycling, ensuring that toxic components are isolated or neutralised [169]. Regulatory incentives stimulate sustainable disposal practices, reduce the environmental footprint of PV technologies, and ensure manufacturers stay on the right track through strict monitoring. Along with regulation, environmental responsibility implies proactive innovation and sustainable design choices [12, 24]. The design of lead‐free perovskites, such as tin‐ and bismuth‐based variants, is under investigation to ensure high efficiency without posing risks as poisonous alternatives [16]. Other technologies that support the environment include passivation layers, low‐temperature fabrication processes, and modular designs that favor recycling. Continuous monitoring of air, soil, and water surrounding production and deployment sites, and detecting contamination at the earliest stages, is also important and enhances transparency with the local people [12]. A balance between technology development and ecological and social responsibility, coupled with strict adherence to occupational safety standards and the safe utilisation of hazardous materials is critical to ensuring the sustainability of the PV industry.

### 7.1 Capacity Building, Training, and Energy Affordability in Photovoltaic Deployment

The effective implementation of PV technologies is not only technologically novel but also relies on human capacity, stakeholder involvement, and equal access to energy [149]. The development of the required skills, knowledge, and social structures is essential to ensure the practical implementation of the environmental and economic advantages of PV systems. The sustainable adoption of PV technologies is based on capacity building and training [170]. The successful deployment depends on manufacturers, technical staff, workers, and local people being equipped with knowledge of how to use, maintain, and safely manage PV systems [170]. In the case of communities, capacity‐building activities include participation (involving the community in decision‐making), increasing awareness regarding the usefulness and possible dangers of PV deployment, and empowering the residents to contribute to the community. These interactions will foster local ownership and trust, ensuring that PV projects align with community priorities and cultural practices. Specialised training is needed to ensure that production is safe, efficient, and environmentally responsible for manufacturers and technical personnel. The manufacturing of PV modules, especially lead‐based PSCs, involves handling sensitive chemicals and hazardous materials [171]. Occupational safety, chemical handling, quality control, and environmental management practices should be provided to workers to reduce health hazards and ensure high‐quality production [172]. Continuous education programs also allow workers to adapt to changing technologies and ensure compliance with safety and environmental standards.

**FIGURE 11:**
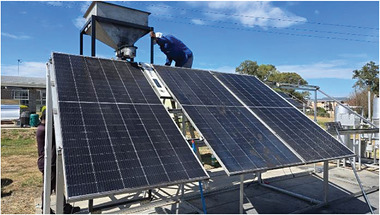
Capacity building, training, and energy affordability in photovoltaic deployment.

As illustrated in Figure 11, investing in capacity building and specialised training significantly lowers the hidden “soft costs” of PV systems, such as delays, poor system design, and installation flaws [173]. When regulators, technicians, financiers, and community leaders are properly trained, projects are expedited, systems perform better, and maintenance costs decrease over time. Skilled installers ensure high‐quality work, while informed policymakers and financial professionals streamline approvals and improve access to funding. Ultimately, these efforts make solar energy more affordable, reliable, and accessible, especially for low‐ and moderate‐income households.

The potential cost‐effectiveness and social benefits of PV are key advantages driving their emergence and implementation in the PV market [174]. Although PV systems have the potential to reduce future energy expenditures, the initial costs of installation and maintenance are high, limiting the rights of marginalized communities [175]. The mechanisms used to finance PV technologies, such as micro‐loans, pay‐as‐you‐go, subsidies, or community‐owned energy cooperatives, make PVs affordable and inclusive [176]. Cheap energy increases living standards, household and small‐business productivity, and offers more people access to economic and educational opportunities [177]. PV value chain on a larger scale is also fundamental in economic empowerment and energy access. A well‐organised local value chain that can create jobs, spur local economies and minimise reliance on imports can be achieved by sourcing raw materials, manufacturing modules, installing and maintaining them locally. Coupling the employment of local people, technical education, and entrepreneurship into PV implementation and deployment plans will help communities achieve long‐term socio‐economic benefits and access to clean energy.

Sustainable deployment of PV, in a nutshell, means that technology, human capital, and socio‐economic structures need to be considered synergistically [178]. Capacity building helps manufacturers, workers, and communities gain the knowledge and skills to adopt PV safely and effectively, and the affordability of energy and inclusive value chains ensure the benefits of renewable energy are shared fairly [179]. Combined, these strategies promote a renewable energy transition that not only seems environmentally sustainable but also socially inclusive and economically empowering. Further improvement can be achieved by integrating stronger interdisciplinary collaboration among materials scientists, policymakers, and social scientists to ensure that photovoltaic deployment is technologically efficient and environmentally benign. Further, continuous monitoring frameworks and adaptive governance mechanisms are needed to respond to emerging risks and technological advancements in the renewable energy landscape.

## 8 Future Perspectives

Global clean energy transitions in the power and transportation sectors hinge upon the development of improved technologies [180]. The PSCs based on lead are at a crossroad, both in terms of technological potential and moral accountability. This emerging technology can significantly accelerate energy access worldwide and help decarbonise the world. Still, its development cannot be considered outside the context of the social and environmental consequences of lead use [16]. This does not require a technical challenge, but is a challenge of how well justice and equity can be incorporated into the energy transition. Generally, the PSC revolution has the potential to introduce environmental vulnerability into vulnerable regions. Energy justice requires that communities gain affordable energy and environmental protection, without one at the expense of the other [181]. Nevertheless, through intentional management, circular economy, and community‐oriented implementation, PSCs can be essential in achieving climate objectives and improving social justice [182]. Although PSCs play a key role in the growth of renewable energy, its projected commersialisation potentially causes localised lead pollution exacerbated by poorly developed recycling protocols, which further introduce new environmental justices. Besides, excessive cautionary limitations or bans on PSC use due to toxicity will delay access to clean energy by hundreds of millions, and thus increasing energy inequality. Consequently, justice‐by‐design integration is expected to adopt a globalised approach to ensure the principles of justice are ingrained throughout the PSC value chain [183]. Between 2025 and 2028, the foundation phase establishes the International Perovskite Stewardship Council (IPSC) certification standards, which are justice‐weighted, and pilot‐scale deployment is implemented only in areas with sufficient circular‐economy infrastructure [184] (cf. Table 5). The scaling phase (2029–2035) will involve blockchain‐based lead management, on‐site recycling micro factories, community‐based monitoring, and safety‐by‐design innovation. Workers in the fossil fuel industry can be shifted to perovskite recycling using Just Transition bonds. In 2036–2040, over 99% of lead is recovered, community energy cooperatives manage a large share of small‐scale installations, and PSCs supply electricity to hitherto underserved communities without posing environmental and public health risks.

**TABLE 5:** A practical 2025–2040 roadmap for lead‐based perovskite solar cells, outlining key targets for efficiency, stability, large‐scale production, and responsible environmental management.

Phase	Years	Global PSC Capacity (GW)	Lead Containment Reliability	Lead Recovery Rate	Governance and Certification	Social and Justice Targets	Refs.
Foundation	2025–2028	1–10	≥95–99% encapsulation compliance; <5 µg/L leachate	60%–80%	Establish IPSC; justice‐weighted certification; blockchain lead tracking in pilots	≥40% community participation in pilots; ≥5000 workers retrained	[191]
Scaling	2029–2035	20–250	≥99% contamination risk reduction	85%–95%	Harmonized global standards (≥40 countries); mandatory EPR; AI monitoring ≥70% systems	≥150M underserved people electrified; ≥25% community ownership in Global South; ≥100 000 fossil workers transitioned	[192]
Maturity	2036–2040	400–800	≥99.5% containment; <1 µg/L field release	≥98%–99% closed‐loop recycling	Full global IPSC compliance; justice‐weighted carbon credits	≥300M people electrified equitably; ≥50–60% small‐scale systems locally owned	[24, 193]

As PSC technology nears commercialisation, governance structures should work at several levels on a global scale, where standards should be synchronized and national capabilities taken into consideration [185]. Also, at the regional level, where implementation frameworks are to be developed, co‐created, and supported locally through community oversight and technical and financial resources [186]. Multi‐barrier encapsulation, decentralised recycling systems, early‐warning monitoring, and ongoing research and developer work on lead‐free alternatives are necessary to integrate technology. Economic processes, including extended producer responsibility, justice‐minded funds, green premiums, and insurance instruments are essential to ensure accountability and financial sustainability. Social innovations such as participative technology evaluation, co‐production of knowledge, models of energy sovereignty, transparent digital platforms, etc., enable communities to be active participants rather than passive recipients. Sector‐based roadmaps focus on a customised approach [27]. PV systems built into building envelopes (building‐integrated photovoltaics (BIPV)) must include lead‐sequestration and obligatory recyclability [187, 188], and be adopted in social housing at the start of the 2030s. Rural off‐grid electrification in Sub‐Saharan Africa, Indo‐pacific and Latin America must focus on fully encapsulated regional recycling centers, community health training, and locally built and owned microgrids. Agrivoltaic systems in climatically sensitive agricultural areas require impenetrable fences, soil‐resistant buffer plants, built‐in surveillance, and specific actions to enhance energy and individual produce [189, 190]. Efficiency and cost are not the only measures of PSC deployment success. Distributional justice, procedural equity, vulnerability reduction, restorative capacity, circularity, and knowledge justice are all measures of the moral and social aspects of technology deployment. A risk management system that combines preventive encapsulation with prudent siting, containment, remediation, and restoration, along with adaptive governance in tiers, will guarantee safety and resilience.

Toward this end, AI can become an essential enabler, predicting degradation patterns, improving recycling networks, anticipating early system failures, modeling the outcomes of justice, and enabling participatory decision‐making [194]. Lead‐based PSC technology is not yet a viable option, but it is a point of divergence in society. They have a three‐ to five‐year window of opportunity to shape their growth responsibly before large‐scale commercialisation. Thus, researchers, policymakers, industry, and civil society are required to collaborate to ensure that justice is prioritised alongside efficiency, develop feasible governance structures, and build the infrastructure of the circular economy [12]. They should also make communities equal partners in their co‐design and development, and sustain pluralism in innovation by not abandoning research into lead‐free options [27]. Successful deployment of PSCs will not be measured by efficiency or market share; it will be the ability to increase access to clean energy without diminishing the health, dignity, or sovereignty of vulnerable communities [73].

## 9 Conclusions

The continued expansion of solar PV deployment is constrained by significant factors including energy justice, environmental sustainability, inclusivity and equity. The potential of PSCs based on lead is remarkable because they can accelerate energy availability and decarbonisation, but this potential comes with an even higher ethical cost. PSCs will continue to pose potential new hazards and perpetuate inequality. There are four interconnected principles of a fair transition: deployment should be localised, the concept of justice should be integrated, it should be grounded in circular economy principles with high lead recovery efficiency, and it should enable communities to become active participants in the green energy transition. Technical measures such as encapsulation and recycling are essential, but they must be supplemented by good governance and a fair political climate, involvement in decision‐making, and accountability. It is important to note that transformative clean energy technologies can only be attained when social and environmental impacts are envisioned, risks are managed, and benefits are distributed equally. The global clean energy transition represents an urgent and multidimensional challenge as the world strives to limit global warming to 1.5°C and achieve net‐zero carbon emissions by 2050. This review contributes to theory and practice. Theoretically, it integrates dimensions of lifecycle operational challenges, material scarcity, and technological maturity. In practice, this study informs deployment strategies and technology selection, highlighting critical bottlenecks in commercialisation pathways for PSC technology. Grid integration strategies, including emerging AI‐driven demand response, virtual power plants, and inter‐regional transmission expansion, may offer practical solutions for future PSC deployment in sub‐Saharan Africa, Indo‐Pacific regions, Latin America and beyond. The real test for the success of the PSC technology will be to offer safe, equitable access to energy, support human dignity, and serve justice. The perovskite revolution will either be an example of responsible innovation or the repeat of the injustices of previous technological introductions. Justice‐by‐design is not a choice; it is a necessity toward a sustainable and just clean energy future and acceleration of global efforts to achieve net‐zero emissions by 2050.

## Author Contributions

**Benjamin K. Korir**: investigation, writing – original draft, methodology, formal analysis. **George G. Njema**: investigation, writing – original draft, methodology, data curation. **Thembinkosi Donald Malevu**: writing – original draft, validation, writing – review and editing. **Joshua K. Kibet**: conceptualization, investigation, methodology, supervision, writing – review and editing. **Mojeed A. Agoro**: investigation, writing – original draft, methodology, validation, writing – review and editing.

## Ethics Statement

The author has nothing to report

## Consent

This article has the consent of all the authors.

## Conflicts of Interest

The authors declare no conflicts of interest.

## Data Availability

The data that support the findings of this study are available from the corresponding author upon reasonable request.
